# A fusion data security protection scheme for sensitive E-documents in the open network environment

**DOI:** 10.1371/journal.pone.0258464

**Published:** 2021-12-15

**Authors:** Lei Liu, Mingwei Cao, Yeguo Sun

**Affiliations:** 1 School of Computer Science, Huainan Normal University, Huainan, China; 2 School of Computer Science and Information Engineering, Hefei University of Technology, Hefei, China; 3 Anhui Province Key Laboratory of Industry Safety and Emergency Technology, Hefei, China; 4 School of Finance and Mathematics, Huainan Normal University, Huainan, China; Civil Aviation University of China, CHINA

## Abstract

E-documents are carriers of sensitive data, and their security in the open network environment has always been a common problem with the field of data security. Based on the use of encryption schemes to construct secure access control, this paper proposes a fusion data security protection scheme. This scheme realizes the safe storage of data and keys by designing a hybrid symmetric encryption algorithm, a data security deletion algorithm, and a key separation storage method. The scheme also uses file filter driver technology to design a user operation state monitoring method to realize real-time monitoring of user access behavior. In addition, this paper designs and implements a prototype system. Through the verification and analysis of its usability and security, it is proved that the solution can meet the data security protection requirements of sensitive E-documents in the open network environment.

## 1 Introduction

With the rapid development of information technology, people pay increasing attention to data security [1, 2]. Especially in some special application scenarios, a more open network environment is needed, and data scattered on the end nodes impose higher requirements on the traditional centralized data security sharing scheme. As a kind of data carrier, electronic documents (E-documents) are different from unstructured data in structure, integrity and storage form diversity [3], but the traditional data security protection scheme remains applicable after adjustment. This paper focuses on the data protection of sensitive E-documents in storage and access control policies in the open network environment, and we propose a fusion scheme to realize the security of the whole access process of sensitive E-documents.

The remainder of this paper is organized as follows. Section 2 briefly explains the related work. Our scheme is detailed in Section 3. Section 4 analyses the availability and security of our scheme. Section 5 discusses the performance evaluation results and system implementation. Finally, Section 6 concludes this paper.

## 2 Related work

Data security includes data transmission security, data storage security and data access security [2]. This paper focuses on the latter two aspects.

At present, data security protection technologies are mainly based on cryptography and are represented by data encryption algorithms and data access control strategies. These technologies focus on the security issues of data storage, distribution, and authorization. Among them, classic data encryption algorithms are divided into symmetric encryption algorithms and asymmetric encryption algorithms [4, 5]. To solve the problem of data sharing in the open network environment, many encryption schemes have been proposed, such as the proxy re-encryption algorithm (PRE) [6] and the identity-based encryption algorithm (IBE) [7, 8]. In recent years, an increasing number of researchers have paid attention to attribute-based encryption (ABE) [9, 10]. Sahai and Waters first proposed the notion of ABE in [11], which achieved one-to-many secure services based on public key encryption, and it ensured efficient encrypted access policy. Indeed, many ABE schemes have been proposed, and Goyal et al. [12] further defined the concept of ABE. In general, ABE schemes are classified into two categories. One kind is key policy attribute-based encryption (KP-ABE) [13, 14], in which the data user’s secret key and ciphertext are relevant to access the policy and attribute set, respectively. The other kind is cipher-text policy attribute-based encryption (CP-ABE) [15], which was first put forward by Bethencourt et al. in [16] and was proved to be safe under the general group model. In a CP-ABE scheme, the data user’s secret key is related to the attribute set, and ciphertext is related to the specific access policy [17–19]. The ABE algorithm can control the user’s decryption ability flexibly and realize the fine-grained access control of sensitive data cipher-text. Consequently, it can meet the needs of sensitive E-documents in both secure storage and secure distribution.

Regarding data access control policy, there are three classic traditional access control models: discretionary access control (DAC), mandatory access control (MAC) and role-based access control (RBAC) [20, 21]. Among them, there are more studies based on RBAC. Cai et al. proposed the model MR-RBAC [22] based on role extension, which improved the role hierarchy and permission inheritance relationship of the traditional model by introducing the minimum role set between the role set and permission set. Yu et al. proposed the property and trust-based access control model ATRBAC [23], which optimizes the dynamic authorization management process. In [24], Rao proposed the role recommender-RBAC, in which a role recommendation model is introduced for the RBAC system to optimize user-role assignments based on user behavior patterns. In recent years, the attribute-based access control model (ABAC) [25–27] can not only meet the fine-grained access control requirements of sensitive data security management systems but also support the large-scale dynamic expansion of users, so it is an ideal access control scheme.

The above research results can meet the security protection requirements for sensitive E-documents in the process of storage, distribution, dynamic authorization, and access control. However, these schemes are mainly for the centralized management of data. Among them, the data, the key, and the authorization management services are stored and processed on the server, which is responsible for the entire data security business of the whole framework. For example, in some application scenarios, the key system configuration documents and database documents must be stored on the client, which results in decentralization and independence. Consequently, it is impossible to improve the security of the server to meet the security requirements of sensitive E-documents so that they are transferred to the client. In this regard, the focus of the sensitive E-document access application scenario is not the centralized and secure storage and distribution of sensitive data but how to solve the problems of sensitive data in the client, such as secure storage, key security management, access control, and user operation control.

**Fig 1:**
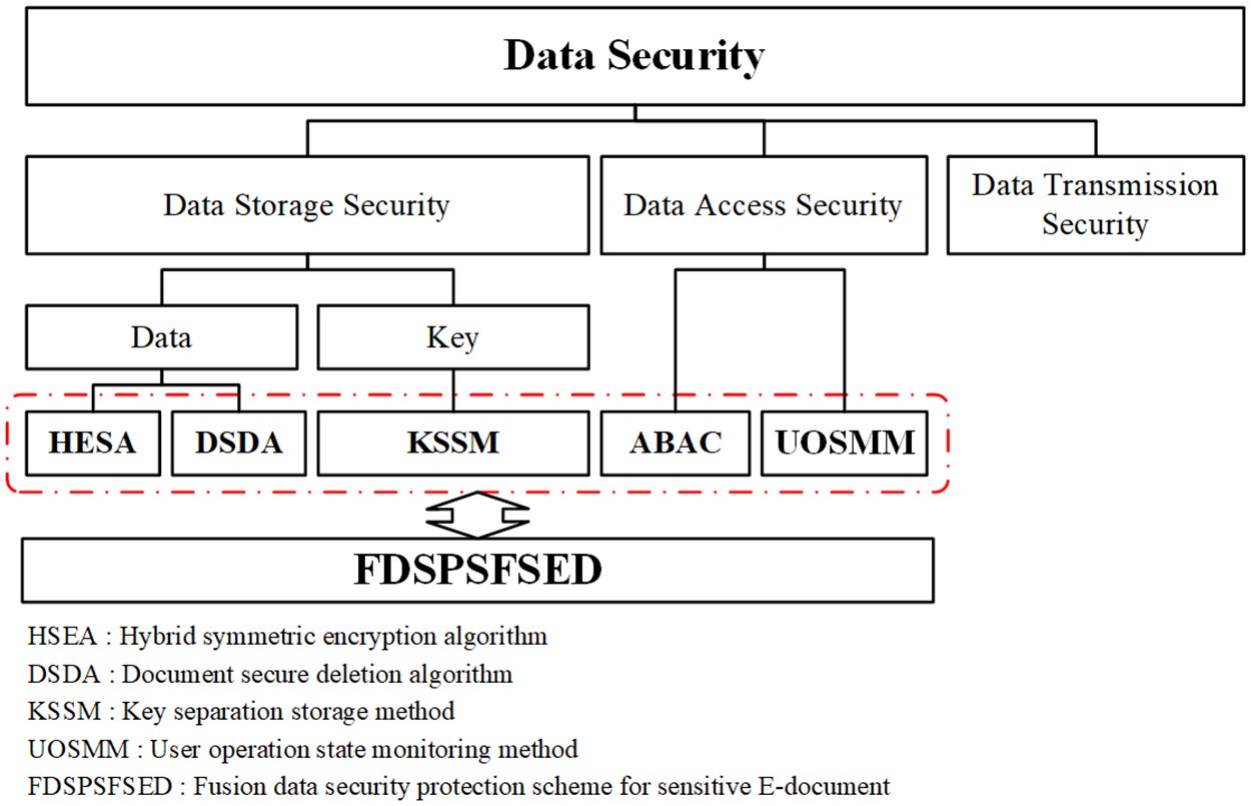
Scheme framework.

Based on the above analysis, we propose a fusion data security protection scheme for sensitive E-documents in the open network environment (FDSPSFSED). As shown in Fig 1, the hybrid symmetric encryption algorithm (HSEA), the document secure document deletion algorithm (DSDA) and the key separation storage method are designed to effectively improve the security of data storage, and the user operation state monitoring method (UOSMM) is introduced with the ABAC to realize flexible user access control. The detailed security proof and implementation results demonstrate the security and practicality of our proposal.

The primary advantages of the scheme are summarized as follows:

- We propose the KSSM to store the different components of the encryption key in the server and the client and rely on the periodic key update method and dynamic key generation method, which makes it difficult for malicious users to obtain the completed key, thus effectively improving the security of the key.
- In terms of data security storage, we propose the HSEA and the DSDA. First, the HSEA is based on two traditional data encryption algorithms: stream encryption and block encryption are used to encrypt the document data, and the efficiency and security of the algorithm are considered. Second, the DSDA can effectively solve the problem of illegal data recovery through multiple rewriting and coverage methods.
- In terms of the data access control policy, we design an access control scheme based on ABAC and UOSMM. On the one hand, by using ABE as the key and using HSEA for E-documents, it not only realizes lightweight security access control policy but also reduces the amount of data decryption calculation; on the other hand, the user operation behavior information is collected in real time by the client monitoring program and summarized to the server, which can realize the global online monitoring of user access status.
- Through the design of a fully functional client program, this scheme shares the business load of the server by breaking up the whole into parts, which effectively improves the overall efficiency of the system.

## 3 Our scheme

### 3.1 System model

This section presents the overall framework of our scheme and the construction of the solution.

#### 3.1.1 System framework

**Fig 2:**
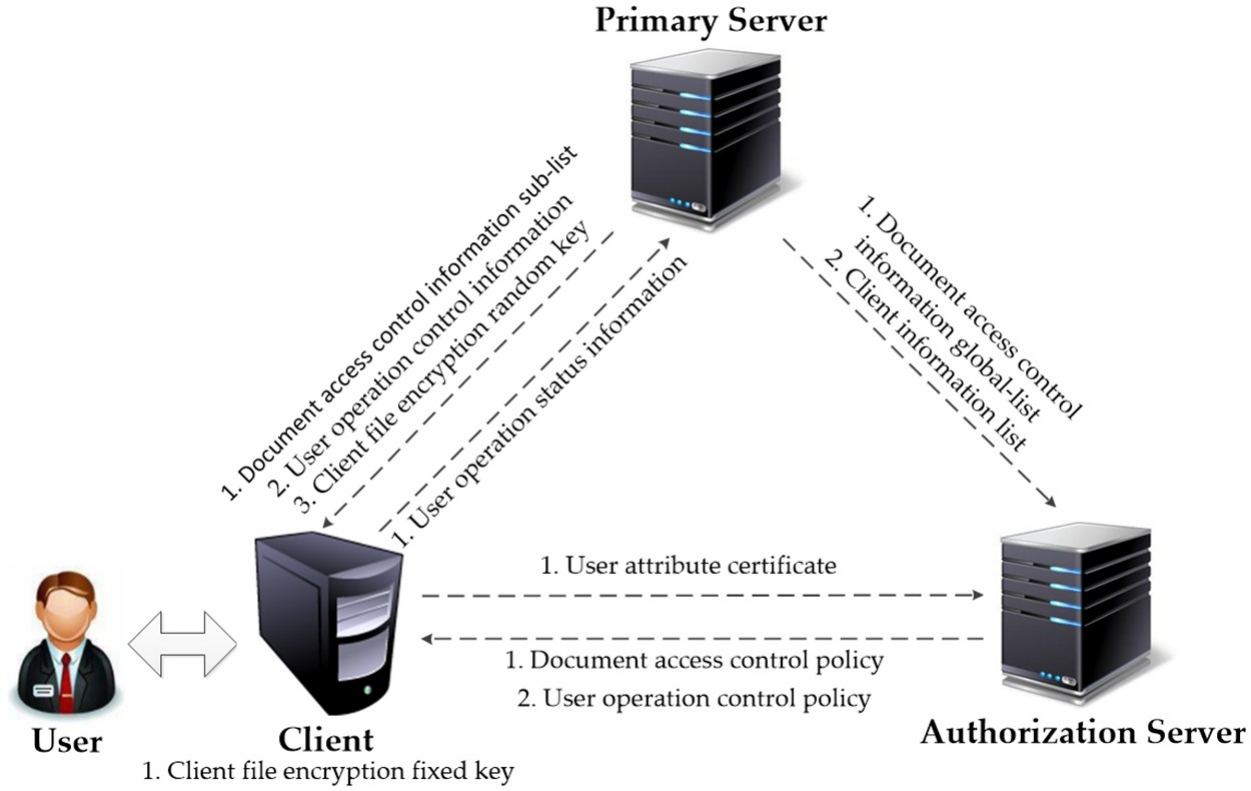
System framework.

In Fig 2, the system framework contains the following three entities.

*3*.*1*.*1*.*1 Primary server*. The primary server is mainly responsible for verifying the user’s identity information, managing the client information and sensitive E-document information, concurrently maintaining and updating the encryption key of the sensitive E-document, and displaying the user’s real-time operational status. It maintains two tables: one is the client information list (List_c), and the other is the document access control information list (List_f).

*3*.*1*.*1*.*1*.*1 List_c*. {Client number, Client basic information}. The client number is assigned by the primary server when the client is initially registered, which is the unique identification of the client; the basic information of the client includes the client registration time, client model, client operating system, and client purpose.

*3*.*1*.*1*.*1*.*2 List_f*. {Document number, Document basic information, Document attribute set, Document sensitivity level}. The basic document information includes the document purpose, document creation time, document size, etc.; the document attribute set is a set of attribute sets describing document characteristics and is mainly used for threshold comparison with user attributes to achieve fine-grained access control of documents. The list of document access control information is divided into a global list and sublist. Among them, the global list is the E-document information of all clients saved by the primary server, and the sublist is the E-document information saved by each client. The document sensitivity level (*Level*) is divided into Level_1, Level_2 and Level_3, where Level_1 refers to the highest sensitivity level of the E-document, Level_2 refers to the intermediate sensitivity level, and Level_3 refers to the general sensitivity level. According to the different document sensitivity levels, the corresponding user operation control authority is also different. The information of the user operation control authority includes permission, time limit, condition, etc.

*3*.*1*.*1*.*2 Authorization server*. The authorization server is mainly responsible for registering and authorizing users, generating user attribute certificates, registering and authorizing clients, managing the public or private keys of clients, managing the operational authority of sensitive E-documents, and creating the user operation control policy.

*3*.*1*.*1*.*3 Client*. The main work of the client is divided into three aspects. First, it is responsible for encrypting or decrypting sensitive E-documents. Second, it is responsible for safely deleting sensitive electronic documents. Third, it is responsible for real-time monitoring of the user’s operational behavior according to the user operation control strategy and returning the information to the primary server.

### 3.2 Security model

This section mainly introduces the detailed implementation process of the scheme. Table 1 lists the entity objects involved in the security model, and the parameter objects are listed in Table 2.

**Table 1:** Entity definition.

Notations	Description
Sm	Primary server
Sr	Authorization server
Client	End node
User	End node user
File	E-Document

**Table 2:** Parameter definition.

Notations	Description
PKc	Public key
SKc	Private key
Kc1	Fixed part of private key
Kc2	Random part of private key
Au	User attribute certificate
Ac	Document attribute set
IDu	User information
IDc	Client information
IDf	File information
List_f_(a/s)	Access control information list (global list and sub list)
List_c	Client information list
Cf	Ciphertext of file
E_X_(y)	Encrypt y with key x
D_X_(y)	Decrypt y with key x
Ru	User operation control policy

#### 3.2.1 Concrete construction of FDSPSFSED

1. **Initialization**
  (a) In the initialization phase, the primary server monitors the connection status with the authorization server and each client and sends the latest *List_f_a* and *List_c* to the authorization server for an update.
Sm→Sr:List_f_a∥List_c (1)
  (b) If a new client is monitored by the system, the client must complete the registration application. First, the client sends its *IDc* to the primary server for authentication and registration applications.
Client→Sm:IDc∥req(authorization) (2)
  (c) If the verification is successful, the primary server sends the *IDc* to the authorization server. The authorization server generates the *SKc* and *PKc* of the client and sends the *SKc* to the primary server and the *PKc* to the client.
$$\left\{\begin{array}{c} Sm\to Sr:IDc\\ S\to Client:SKc,Sr\to Sm:PKc \end{array}\right.$$ (3)
  (d) The primary server first generates *List_f_s* and *Kc2* and then uses *PKc* to encrypt it and send it to the client.
$$Sm\to CLient:E_{PKc}(List\_f\_s∥Kc2)$$ (4)
  (e) The client encrypts the E-documents in *List_f_s* according to *Kc1* and *Kc2*. Among them, *Kc1* is the MD5 value obtained by processing the client CPU number, hard disk number, network card MAC address, and other information through a specific algorithm. The encryption algorithm adopts the HESA that combines RC4 and AES. See Section 3.2.2 for details.
$$Cf=Client:E_{Kc1+Kc2}(File,\left\{File\in List\_f\_s\right\})$$ (5)
2. **User registration**
  (a) The user applies for registration to the primary server, and the information sent together also includes *IDu* and *IDc*.
$$User\to Sm:req\left(register\right)∥IDu∥IDc$$ (6)
  (b) After receiving the registration application, the primary server first verifies the *IDc*; that is, the user must be on the registered client to apply for registration. If it passes, the master server will send the *IDu* to the authorization server; otherwise, access will be rejected.
$$\left\{\begin{array}{c} Sm\to Sr:IDu,IDc=true\\ ⊥,Refuse\;application \end{array}\right.$$ (7)
  (c) According to the basic information of the user, the authorization server issues the *Au* to the user. The content of *Au* includes not only the general certificate but also the user attribute set, which is composed of multiple attribute values that can describe the user’s role.
Sr→User:Au (8)
3. **User requests access to documents**
  (a) Before the user accesses the document, the primary server must verify whether the user can log into the authorized client. If it passes, the validity verification of the user attribute certificate will continue; otherwise, access will be rejected.
$$\left\{\begin{array}{c} Sm\to Sr:Au,\;(IDc=true)\cap (IDu=true)\\ ⊥,\;Refuse\;application \end{array}\right.$$ (9)
  (b) The authorization server is responsible for verifying the validity of *Au*. If it passes, the system continues to wait for the user’s document access application; otherwise, access will be rejected.
$$\left\{\begin{array}{c} 1,\;Au=true\\ ⊥,\;Refuse\;application \end{array}\right.$$ (10)
  (c) The user applies for document access. The client that the user logs in sends the request containing *IDc*, *IDf*, and *Kc2* to the primary server.
User→Client:req(IDf) (11)
Client→Sm:req(IDc∥IDf∥Kc2) (12)
  (d) The primary server first verifies the correspondence between the client and the document according to *List_f_a* to verify whether the document to be accessed is on the current client. If it passes, the primary server sends the client file *Kc2* to the authorization service; otherwise, access will be rejected.
$$\left\{\begin{array}{c} Sm\to Sr:Kc2,\;(IDc=true)\cap (IDf=true)\\ ⊥,\;Refuse\;application \end{array}\right.$$ (13)
  (e) First, the authorization server generates *Ru* according to the level to be accessed in *List_f_a*. Then, the authorization server encrypts the *Ru*, *Ac*, and *Kc2* with the *PKc* and sends it back to the client.
$$Sr:Ru=\left(IDc∥IDf∥User\_op∥User\_t∥User\_c\right)$$ (14)
$$Sr\to Client:\;D=E_{PKc}(Ru∥Ac∥Kc2)$$ (15)
4. **Document access control**
  After *D* is sent back to the client, the client monitors the user’s access behavior in real time. On the one hand, the client controls the access of E-documents through the document decryption control method and the document operation control method; on the other hand, the client sends the user’s operational status to the main server for remote monitoring.
  *Document decryption control method*.
    (a) The client decrypts the received data, parses *IDc* and *IDf* from *D*, and validates them. If it passes, it continues to wait; otherwise, access will be rejected.
$$Client:D_{SKc}(D)$$ (16)
$$\left\{\begin{array}{c} 1,IDc=true)\cap (IDf=true)\\ ⊥,\;Refuse\;application \end{array}\right.$$ (17)
    (b) According to *Ac* and *Au*, the client checks whether the number of attribute values intersected by the user attribute set and the document attribute set meets *d*. If it passes, the user is allowed access; otherwise, access will be rejected.
$$\left\{\begin{array}{c} 1,\;\left|Ac\cap Au\right|\geq d\\ ⊥,\;Refuse\;application \end{array}\right.$$ (18)
    (c) The client generates *Kc1* and decrypts the document with the HSEA according to *Kc2* to obtain the plain-text file.
$$File=D_{Kc1+Kc2}(Cf)$$ (19)
  *Document operation control method*.
    Through file filter driver technology, the client can monitor the user’s operation behavior in real time and judge whether the user’s use steps (*op*), use time (*t*), and use conditions (*c*) for the document meet the requirements of the *Ru*. If it passes, it continues to monitor; otherwise, access will be rejected.
$$\left\{\begin{array}{c} 1,\;op\in user\_op\cap t\in user\_t\cap c\in user\_c\\ ⊥,\;Refuse\;application \end{array}\right.$$ (20)
5. **End of document access**
  After the user completes the document access, the client applies to the primary server again for encryption. The primary server updates the new *Kc2* to *Kc2’*, and the authorized server sends it back to the client. The client completes the encryption operation of the document by combining *Kc1*. The specific process is as follows.
$$Client\to Sm:req\left(IDc∥IDf∥State\left(over\right)\right)$$ (21)
$$Sm\to Sr:Kc2^{\prime }=Sm:Generate\left(IDc∥IDf\right)$$ (22)
$$Sr\to Client:E_{PKc}\left(Kc2^{\prime }\right)$$ (23)
$$Client:E_{Kc1+Kc2^{\prime }}(File)$$ (24)

#### 3.2.2 Hybrid symmetric encryption algorithm (HESA)

In this paper, the symmetric encryption algorithm is used to encrypt E-documents on the client to protect the security of sensitive data. Users can access data only when they are authenticated, and their attribute sets must meet the requirements of the document attribute sets. However, the document-oriented encryption method is different from the traditional text encryption method, which requires consideration of the data encryption operation at the file system level. The encrypted content is not the plain-text content in the E-document; rather, the document is encrypted as an independent object.

Accordingly, we design the HESA, which makes use of the advantages of RC4 and the AES in stream data and block data. The keys of the two encryption algorithms correspond to *Kc1* and *Kc2*.

This method stores the different parts of the encryption key in the primary server and the client separately, so the client must obtain the *Kc2* part of the key from the primary server for each encryption or decryption operation of the document. Because *Kc2* is randomly generated and regularly updated by the primary server, it is difficult for malicious users to obtain both *Kc1* and *Kc2* at the same time, so this can effectively improve the security of the key.

*Kc1* is calculated by the specific algorithm according to the client CPU, hard disk, network card, and other hardware parameters. *Kc2* is generated and updated regularly by the primary server. In HESA, the different components of the key are stored in the primary server and the client by KSSM so that the client must obtain *Kc2* from the primary server every time the document needs to encrypt or decrypt. *Kc2* is generated randomly by the primary server and updated regularly. It is difficult for malicious users to obtain *Kc1* and *Kc2* at the same time, which can effectively improve the security of the key. The encryption process is as follows.

**Step 1**. Read the document property information.
**Step 2**. Read the plain-text data in blocks 1 MB in size, calculate the MD5 value of the block data, and attach it before the data.
**Step 3**. Determine whether the document is in compressed format. If not, compress the block data; otherwise, execute Step 4.
**Step 4**. Taking *Kc1* as the key, the RC4 algorithm is used to encrypt the data in streams.
**Step 5**. Taking *Kc2* as the key, the AES algorithm is used to encrypt the data in blocks.
**Step 6**. Write block data to the output file in turn.

#### 3.2.3 Document security deletion algorithm (DSDA)

To ensure the data security of E-documents on the client, this study designs the DSDA for E-documents to be deleted on the client. In DSDA, the original text is rewritten and overwritten by modifying the document name, writing random content in the form of a data stream three times, truncating the document to prevent the document from being recovered maliciously, and ultimately achieving the purpose of safe deletion of the document. Experiments show that a document deleted by the DSDA is not easy to recover. Therefore, the DSDA has high security and availability. The algorithm process is as follows.

**Step 1**. Modify the document name to a random string.
**Step 2**. Generate a random integer (r1), and write all r1 to the document in 32 byte data blocks.
**Step 3**. Refresh the data, and close the file.
**Step 4**. Repeat Steps 1–3, generate random integer(r2) in step 2, and write all r2 to the document.
**Step 5**. Repeat Steps 1–3, generate a random integer (r3) in step 2, and write all r3 to the document.
**Step 6**. Truncate the document size to 0.
**Step 7**. Delete the document.

#### 3.2.4 Key separation storage method (KSSM)

Our scheme uses HESA in the client to encrypt and decrypt the E-documents, which can improve the data encryption and decryption rate, but the symmetric encryption algorithm is weak in key security protection. Consequently, we divide the key into two parts, which are generated dynamically by the primary server and the client when encrypting or decrypting the E-documents. *Kc2* is generated by the server according to a specific algorithm, and *Kc1* is generated by the client according to the hardware information of the local device. The method process is as follows. Steps 1–3 are the initial file encryption phase, and Steps 4–7 are the user requests to access the file phase.

**Step 1**. The primary server generates *Kc2* and sends it to the client.
**Step 2**. The client generates *Kc1* according to the local hardware information.
**Step 3**. The client uses HESA to encrypt the *File* according to *Kc1* and *Kc2*.
**Step 4**. The user requests access to the *File*.
**Step 5**. The client requests *Kc2* from the primary server and generates *Kc1*. Concurrently, the client decrypts the File according to the keys *Kc1* and *Kc2*.
**Step 6**. After user access, the client sends the status to the primary server. The primary server updates *Kc2* to *Kc2’* and sends it to the client.
**Step 7**. The client encrypts the *File* according to *Kc1* and *Kc2’*.

#### 3.2.5 User operation state monitoring method (UOSMM)

**Fig 3:**
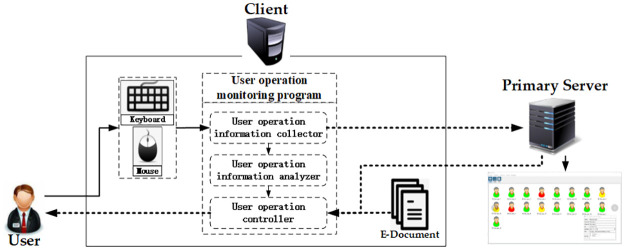
UOSSM framework.

The traditional access control policy is mainly responsible for the user’s access authorization, which cannot monitor and process the user’s subsequent operations. Fig 3 shows that the user operation monitoring program is introduced into the client to analyze the user’s mouse and keyboard operation information to monitor the user’s access behavior in real time and block the user’s violation behavior. Meanwhile, the monitoring program feeds back the state information to the primary server to realize the global monitoring of sensitive E-documents, and the primary server can directly issue orders to the client to control user access behavior.

## 4 Availability and security analysis

In this section, we first design an experiment to verify the availability of FDSPSFSED in three stages of user authentication, user access control, and user operation control, which are the main aspects of sensitive E-documents during the process of user access. Second, we mainly analyze the security of our scheme in data storage.

### 4.1 Availability analysis

The experiment simulates the scenario of a company’s system maintenance department for server maintenance management. All users must access and manage sensitive E-documents through the prototype system. Users are divided into legal users and malicious users. Legal users must have the identity authentication account password and the user attribute certificate issued by the authorization server, while other users are regarded as malicious users.

There are six users in Table 3, and the attribute set in the *Au* includes three values: department, professional level, and working life. The top four users (User_A~User_D) are legal users, and their access behavior should be in the LAN of the company. The other two users (User_E and User_F) are malicious users, and they both obtain User_A’s account information in an illegal way. User_E works in another department of the company and obtains only the account information of User_A. User_F is a noncompany employee who not only obtains the account information of User_A but also forges *Au’* of User_A. The test scheme assumes that the authorization server has reliable security, and the Au cannot be cracked at the content level. Consequently, the prototype system cannot pass the verification of *Au’*.

**Table 3:** User information.

User_Id	User Account Information	Attribute Set (Au)	Remarks
User_A	Id_u_a,Pass_u_a	{Class 1, Middle, Two years}	Legal user
User_B	Id_u_b,Pass_u_b	{Class 1, Middle, Six years}	Legal user
User_C	Id_u_c,Pass_u_c	{Class 2, High, Five years}	Legal user
User_D	Id_u_d,Pass_u_d	{Class 3, Primary, Three years}	Legal user
User_E	Id_u_e,Pass_u_e	{Class 1, Middle, Two years}	Malicious user
User_F	Id_u_f,Pass_u_f	{Class 1, Middle, Two years}	Malicious user

The information of sensitive E-documents to be accessed by users is shown in Table 4. The *Ac* is also the access control policy of sensitive E-documents, including the document attribute value set and the access control threshold value *d*, and the three attribute values in the attribute value set must meet the conditions of {“= Department”, “≥ Professional level”,“≥ Working life”}. The *Level* is set by the system according to the importance of E-documents to determine the *Ru*{document operation authority, allowed access time limit, and authorized client IP range}. In this case, the *Level* of File_A is Level_2, and the *Ru* of File_A is {Read, 8:00–18:00,10.19.185.*}. The *Level* of File_B is Level_3, and the *Ru* is {Read or Update, 8:00–18:00,10.19.185. *}.

**Table 4:** File information.

File_Id	File Information	Access Control Policy (Ac)	Level
File_A	Id_f_a	{Class 3, Middle, Three years}, d = 2	Level_2
File_B	Id_f_b	{Class 1, High, Five years}, d = 2	Level_3

The detailed access behavior of users in the test scheme design is shown in Table 5. Each user initiated an access request for File_A and File_B. Each attribute value (*use_op*, *use_t*, *use_c*) in the operation set has the same control effect, and any of the attribute values will trigger the access interruption scheme when the conditions are not met. Therefore, to reduce the number of repeated tests, only one of them takes different values as a representative.

**Table 5:** User access file information.

User_Id	File_Id	Operational Details (use_op, use_t, use_c)
User_A	File_A	{Update, 8:50, 10.19.185.140}
	File_A	{Read, 8:50, 10.19.185.140}
	File_B	{Update, 8:50, 10.19.185.140}
	File_B	{Read, 8:50, 10.19.185.140}
User_B	File_A	{Update, 14:30, 10.19.185.70}
	File_A	{Read, 14:30, 10.19.185.70}
	File_B	{Update, 14:30, 10.19.185.70}
	File_B	{Read, 14:30, 10.19.185.70}
User_C	File_A	{Update, 10:00, 10.19.185.100}
	File_A	{Read, 10:00, 10.19.185.100}
	File_B	{Update, 10:00, 10.19.185.100}
	File_B	{Read, 10:00, 10.19.185.100}
User_D	File_A	{Update, 13:00, 10.19.185.25}
	File_A	{Read, 13:00, 10.19.185.25}
	File_B	{Update, 13:00, 10.19.185.25}
	File_B	{Read, 13:00, 10.19.185.25}
User_E	File_A	{Update, 11:50, 10.19.185.110}
	File_A	{Read, 11:50, 10.19.185.110}
	File_B	{Update, 11:50, 10.19.185.110}
	File_B	{Read, 11:50, 10.19.185.110}
User_F	File_A	{Update, 15:00, 214.18.15.120}
	File_A	{Read, 15:00, 214.18.15.120}
	File_B	{Update, 15:00, 214.18.15.120}
	File_B	{Read, 15:00, 214.18.15.120}

Table 6 shows the user access results. The main security protection of the FDSPSFSED includes three stages: user authentication, document access control, and user operation control. Combined with Tables 3–5, the scheme can monitor the whole process at different stages according to the access behavior of the users. Once the user is found to have engaged in illegal behavior, the system immediately blocks the access operation of relevant documents. Consequently, the experimental results show that our scheme in user access control has good availability.

**Table 6:** User access result information.

User_Id	File_Id	User Authentication		Document Access Control (Ac∩Au≥d)	User Operation Control	Result
		Identity Verification	Au Validation			
User_A	File_A	True	True	False	/	/
	File_A	True	True	False	/	/
	File_B	True	True	False	/	/
	File_B	True	True	False	/	/
User_B	File_A	True	True	True	F∩T∩T = False	/
	File_A	True	True	True	T∩T∩T = True	OK
	File_B	True	True	True	T∩T∩T = True	OK
	File_B	True	True	True	T∩T∩T = True	OK
User_C	File_A	True	True	True	F∩T∩T = False	/
	File_A	True	True	True	T∩T∩T = True	OK
	File_B	True	True	True	T∩T∩T = True	OK
	File_B	True	True	True	T∩T∩T = True	OK
User_D	File_A	True	True	False	/	/
	File_A	True	True	False	/	/
	File_B	True	True	True	T∩T∩T = True	OK
	File_B	True	True	True	T∩T∩T = True	OK
User_E	File_A	True	False	/	/	/
	File_A	True	False	/	/	/
	File_B	False	/	/	/	/
	File_B	False	/	/	/	/
User_F	File_A	True	True	True	F∩T∩F = False	/
	File_A	True	True	True	T∩T∩F = False	/
	File_B	False	/	/	/	/
	File_B	False	/	/	/	/

### 4.2 Storage security analysis

In Fig 1, our scheme mainly uses the HESA, the KSSM and the DSDA to realize security storage of data. Since both the AES [28] and RC4 [29] have high security and the HESA combines the advantages of the two algorithms, in this phase, we will not analyze its security.

**Fig 4:**
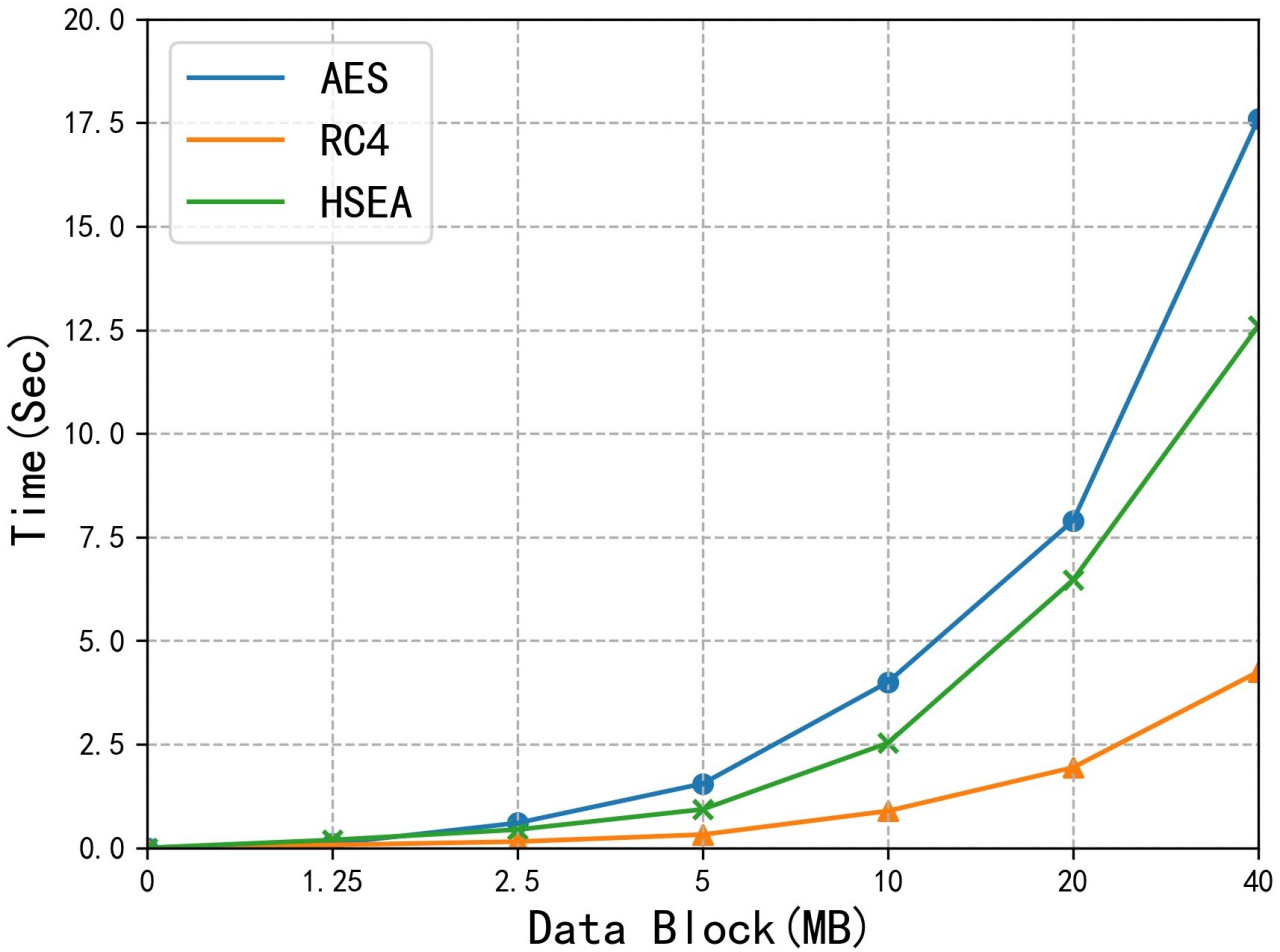
Algorithm rate comparison.

Fig 4 shows the time chart for the encryption/decryption by using AES, RC4 and HSEA. The efficiency of HSEA is better than that of RC4, but it is worse than that of AES. In most cases, since the size of a single E-document is relatively small, rarely exceeding 5 MB, the encryption/decryption rate of the HSEA can meet the requirements of the system.

#### 4.2.1 KSSM security analysis

In this paper, to realize the separate management of the key, the primary server and the client are used to manage *Kc2* and *Kc1*, respectively. Since the client obtains *Kc1* through a special algorithm according to the hardware parameters, it is generated only during the document encryption and decryption operation, so it is difficult for malicious users to obtain the key. In addition, *Kc2* is randomly generated by the primary server and updated regularly, while only the client has the decryption private key for *Kc2*, and *Kc1* will be generated dynamically by the client only when the user gains access, so *Kc1* and *Kc2* will not be stored in the client statically. This scheme ensures that the malicious user cannot obtain the complete key to decrypt the sensitive E-document on the client.

#### 4.2.2 DSDA security analysis

The DSDA is designed to prevent the deleted sensitive E-documents from being recovered maliciously to further improve the storage security of E-documents on the client. In this algorithm, we use multiple rewriting and multiple covering to improve the difficulty of data recovery. In the data recovery experiment, we deleted 100 files with the same content and recovered them using several common data recovery software programs [30–32] during different times of file rewriting operation. In Table 7, [30–32] cannot recover data successfully after three rewrite cycles, and even after only one rewrite, the readability of the recovered files is very low. This can prove that the DSDA has high security in data deletion.

**Table 7:** Data recovery performance.

Recoverable files	Zero time rewriting	One time rewriting	Two times rewriting	Three times rewriting	Four times rewriting
Readable files					
[30]	97	11	1	0	0
	92	2	0	0	0
[31]	90	2	0	0	0
	85	0	0	0	0
[32]	91	5	0	0	0
	89	0	0	0	0

## 5 Evaluation and implementation

A good data security protection scheme must have the characteristics of light weight and high security in data algorithms and must meet the requirements of flexibility and fine-grained data access control while consuming as few system resources as possible. We have already proved in Section 4 that our scheme performs well in data access control. Consequently, in this section, we evaluate our scheme only in terms of performance and system function and then introduce the prototype system based on the FDSPSFSED.

### 5.1 System evaluation

The system evaluation mainly focuses on the analysis of the system business load and computational costs. In this experiment, the software and hardware configuration of the primary server includes Windows Server 2016 (64 bit); CPU: Intel Core i5–8600 CPU @ 3.1 GHz/4.3 GHz; Memory: 16.0 GB. The client’s software and hardware configuration include Windows 10 (64 bit); CPU: Intel Core i3–9100 CPU @ 3.6 GHz/4.2 GHz; Memory: 8.0 GB. In the performance and system function comparison experiment, the methods used are from [33] and [34], which are based on the attribute-based method. We use the Java Pairing-Based Cryptography library (JPBC) to reproduce [33] and [34], and our algorithm uses the data encryption function library OpenSSL (1.1.1c).

**Fig 5:**
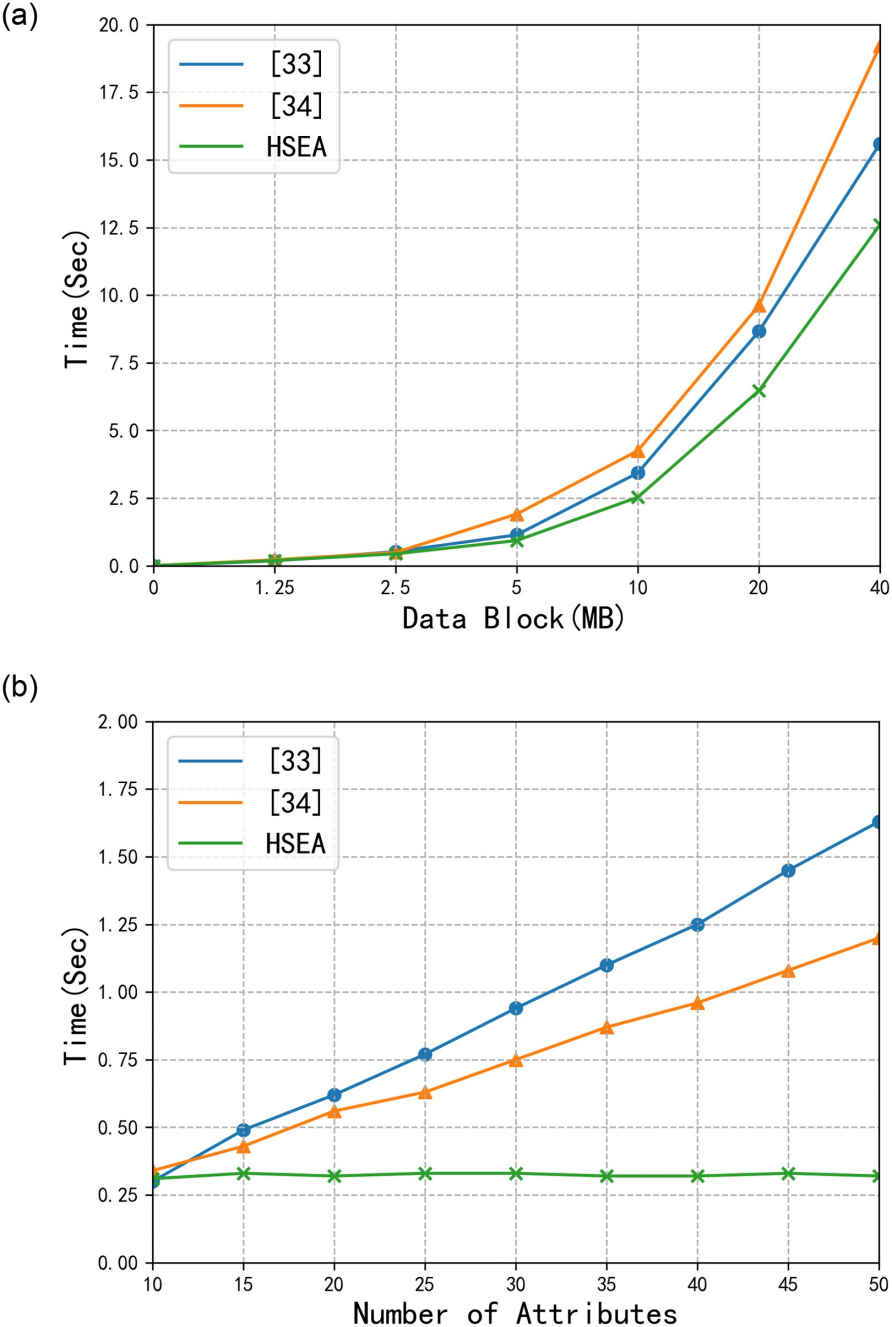
Comparison among [33, 34], and HSEA.

Fig 5(a) shows the time chart for the encryption and decryption by using [33, 34] and the HSEA, respectively. In this experiment, we set the number of attribute values to 8. Combined with the results in Fig 4, we can see that HSEA can not only ensure high data security but also has certain advantages in the data encryption rate. In Fig 5(b), we use data blocks of the same size in the data encryption process but set different numbers of attributes. Since [33] and [34] use attribute-based data encryption algorithms, with the increase in the number of attributes, the time required for data encryption exhibits a linear growth pattern. However, for the HSEA, the number of attributes is used only in the process of data access control, and the symmetric encryption algorithm is used for data encryption, so the increase in the number of attributes does not reduce the data encryption rate.

**Fig 6:**
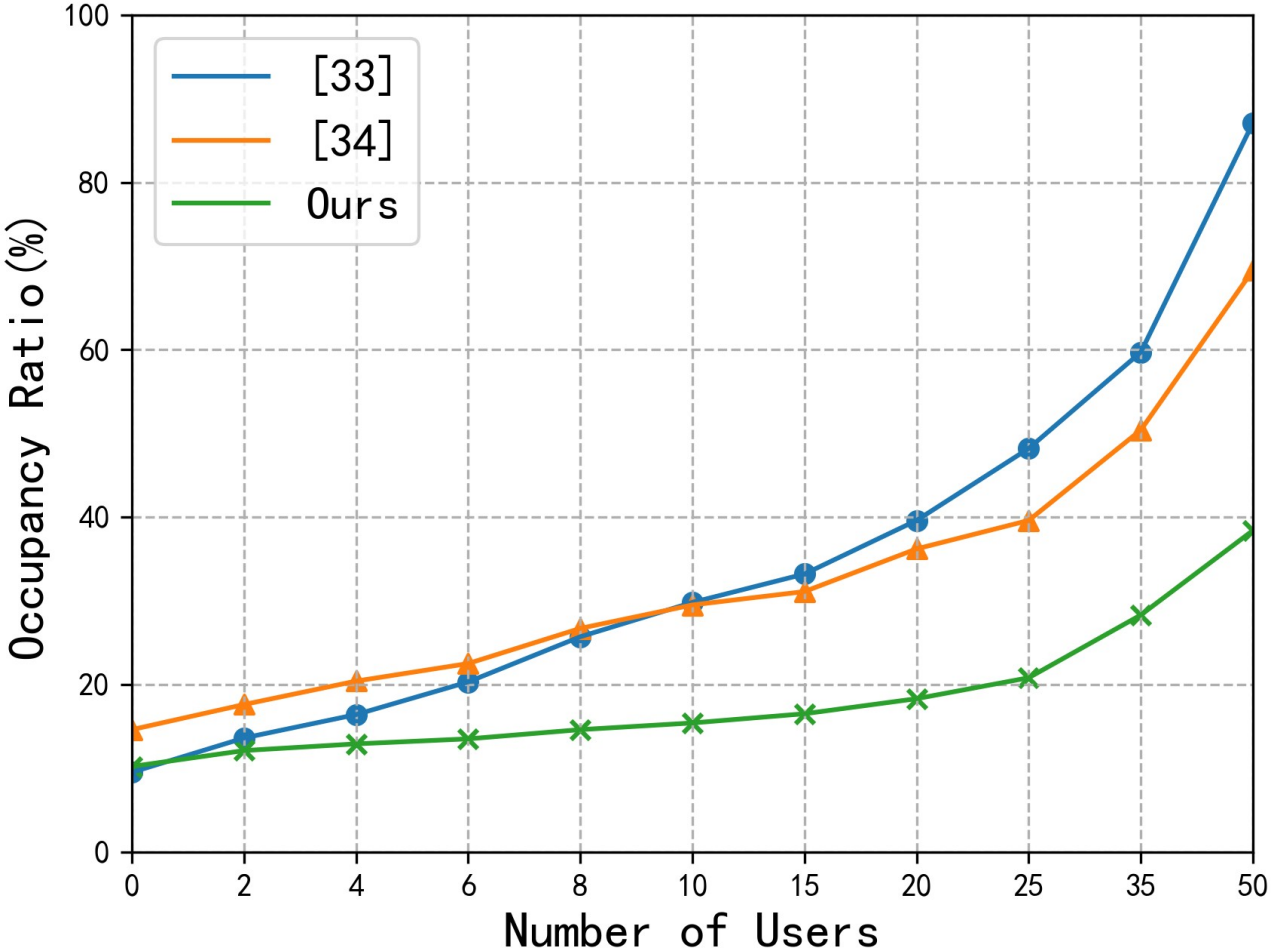
Comparison of network costs.

**Fig 7:**
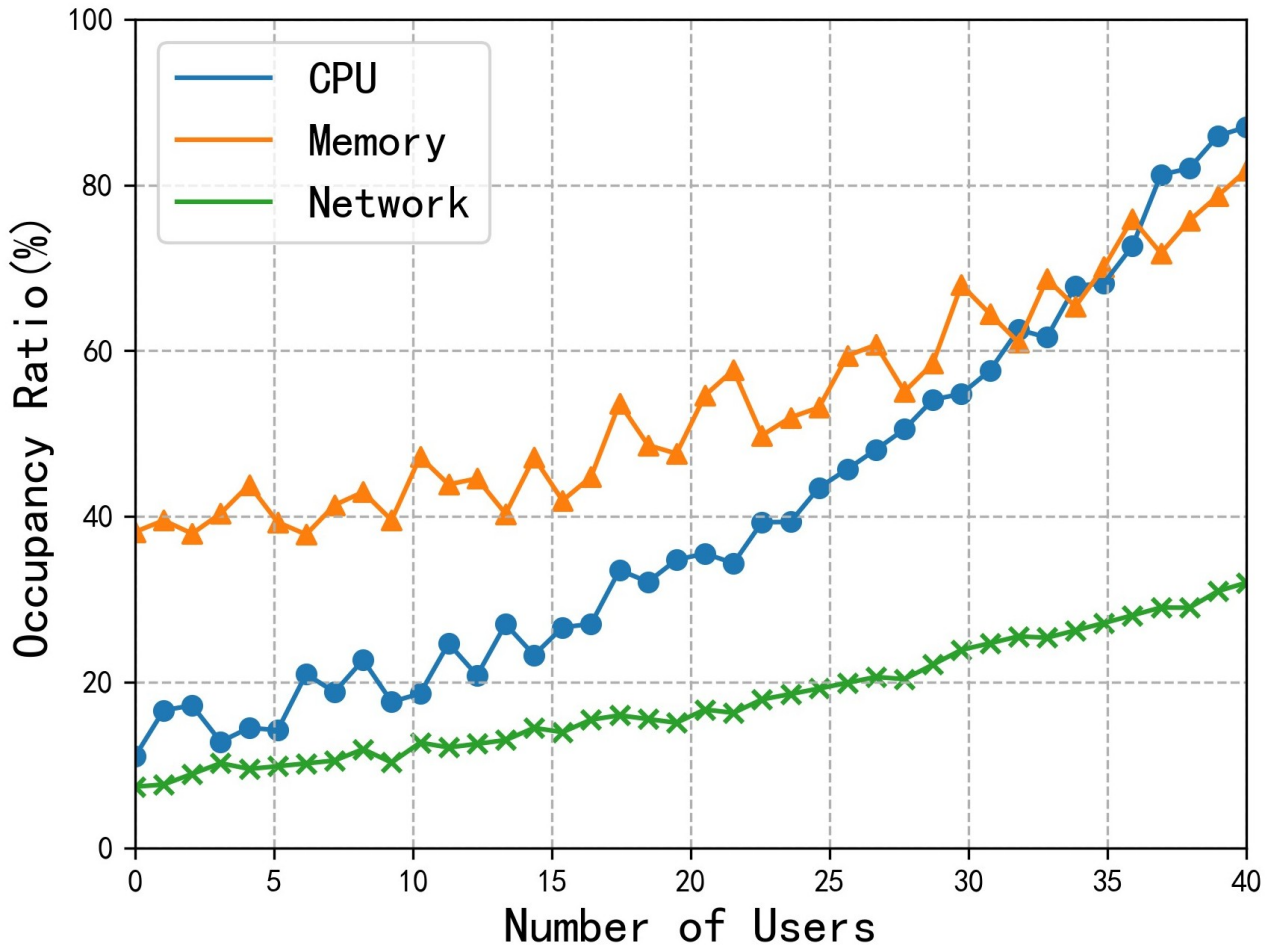
Computational costs of primary server.

In Fig 6, we compare and analyze the network costs of the whole LAN area when using [33, 34] and our scheme. In our solution, the E-documents are stored on the client, and the encryption and decryption of data are performed by the client, so the data transmission of the system is mainly *Kc2*, which greatly reduces the amount of data transmitted. Therefore, the increase in the number of users does not increase the network cost. Fig 7 shows the computational costs of the primary server. As the number of users increases, the costs of CPU, memory, and network also increase. Therefore, under the current hardware configuration of this experiment, the reasonable number of users should be less than 30. In the improvement plan, we can use professional servers and other load balancing equipment to improve the overall performance of the system.

Table 8 presents the function comparison of [33–35] and our scheme in the process of data encryption, data decryption, key management, and data transmission. [35] is based on the traditional PKI method. This indicates that the advantages of our solution on the client side are not obvious. The main reason is that the client not only undertakes the process of data encryption and decryption but also is responsible for part of the key management work. However, on the server side, our solution has obvious advantages. It not only reduces the key management on the server side but also reduces the data transmission pressure on the server side because all E-documents are stored on the client side. Therefore, our solution greatly improves the operational efficiency of the system through the cooperation approach of heavy clients and light servers.

**Table 8:** System function comparison.

	DE			DD			KM			DT			Total		
	PS	AS	C	PS	AS	C	PS	AS	C	PS	AS	C	PS	AS	C
[33]	Y	N	N	N	N	Y	N	Y	N	Y	N	Y	2Y	1Y	2Y
[34]	Y	N	N	N	N	Y	N	Y	N	N	N	Y	1Y	1Y	2Y
[35]	Y	N	Y	N	N	Y	N	Y	N	Y	N	Y	2Y	1Y	3Y
**Ours**	N	N	Y	N	N	Y	N	Y	Y	N	N	N	0Y	1Y	3Y

DE: Data encryption DD: Data decryption KM: Key management DT: Data transmission.

PS: Primary server AS: Authorization server C: Client.

### 5.2 System implementation

**Fig 8:**
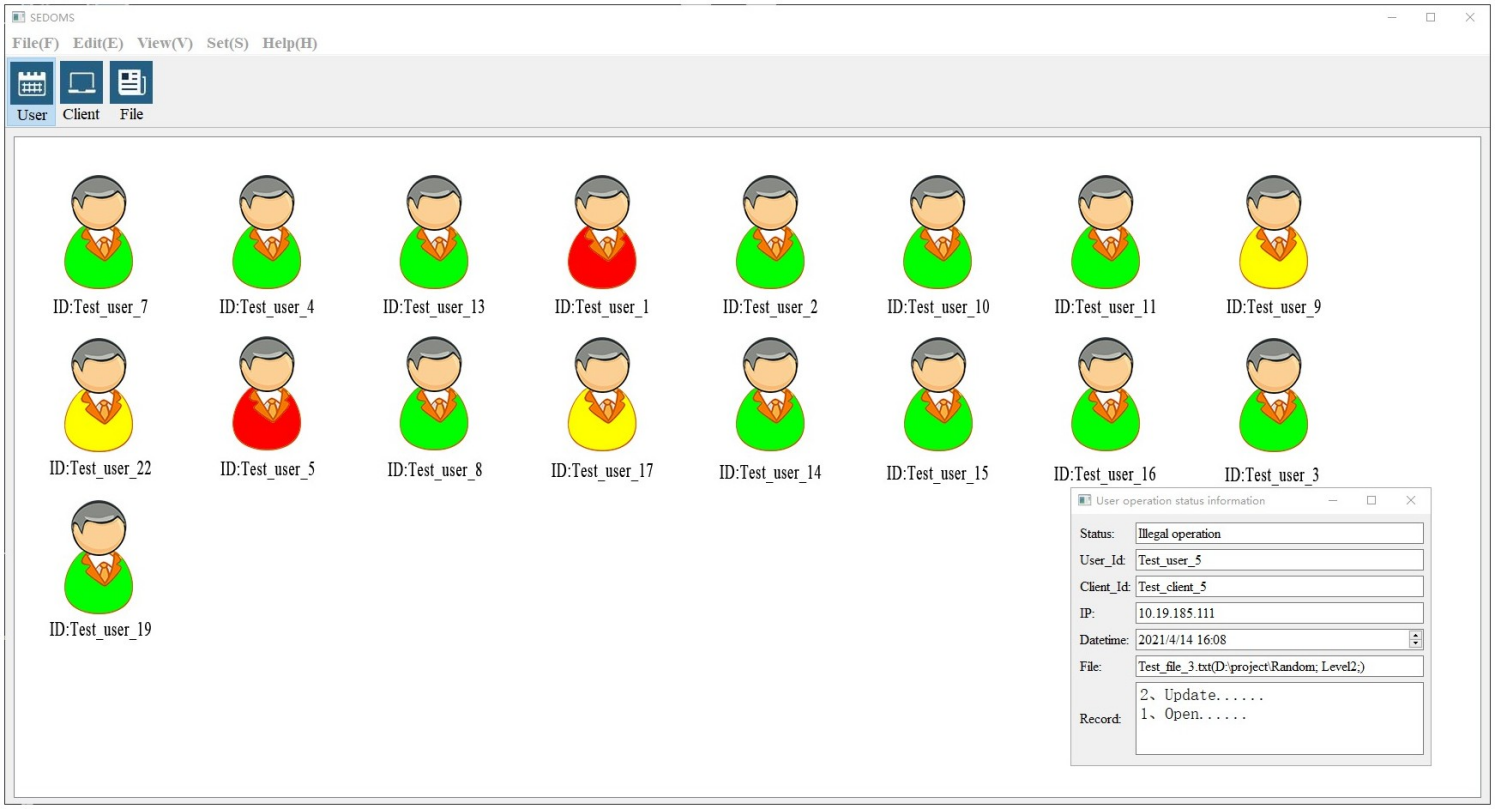
Prototype system.

Fig 8 shows the main monitoring interface of the prototype system based on the FDSPSFSED, which mainly shows the real-time monitoring of the user’s operation behavior [36, 37] by the primary server. The prototype system was developed by Visio Studio (2017), the development framework was QT (5.11.1), and C++ was the programming language.

The prototype system classifies the access status of users and identifies them with different colors. Green represents the pending access status, which is when the user has successfully passed the authentication but has not accessed the E-documents. Yellow represents the compliance access status, which is when the user has successfully passed the authentication and is in the process of accessing the E-documents. Red represents the illegal access status, which is when the user has successfully passed the authentication and is in the process of illegal access to the E-documents. In the test scheme, eight users use different clients to access the sensitive E-documents. The clients monitor the user’s operational behavior in real time and feedback the status information to the primary server, and the server distinguishes the user’s operation status with different colors.

In the results, the system displays that Test_user_7, Test_user_4, Test_user_4, Test_user_13, Test_user_2, Test_user_10, Test_user_11, Test_user_8, Test_user_14, Test_user_15, Test_user_16, Test_user_3, and Test_user_19 are in pending access status; Test_user_9, Test_user_22, and Test_user_17 are in compliance access status; and Test_user_1 and Test_user_5 are in illegal access status. By double-clicking the mark of Test_user_5, we can see detailed information on user access behavior, including the ID and IP of the login client, the occurrence time of illegal operation, document access information, and operational behavior record information. Through this method of data visualization, managers can perform real-time and comprehensive monitoring of the access status of E-documents distributed on different clients.

## 6 Conclusions

This paper proposes a fusion data security protection scheme for E-documents based on the KSSM, the HSEA and the UOSMM. This scheme has the following advantages. First, the system manages the key independently by the server and the client. The key is updated regularly by the server and is generated only when the client encrypts or decrypts the data, which makes it difficult for one malicious user to obtain the complete key. Second, the HSEA and DSDA can not only store the ciphertext of sensitive E-documents but also prevent them from being recovered maliciously. Third, by introducing UOSMM and designing user operational status monitoring programs to control user access behavior, methods such as feedback of status information to the main server, unified monitoring and management of global sensitive E-documents are realized. Fourth, through the design of a client program with complete functions, the main security protection task can be transferred to the client, which can greatly reduce the workload of the primary server and improve the overall efficiency of the system.

In summary, this scheme can provide reliable data security protection for sensitive E-documents in the open network environment. In the future, we will further to investigate and sort out the data security protection requirements in different application scenarios and strive to improve the scheme in terms of improving algorithm speed, access control reliability, and user status monitoring accuracy.

## Abbreviations

The following abbreviations are used in this manuscript:

ABAC: Attribute-Based Access Control Model
ABE: Attribute-Based Encryption Algorithm
AES: Advanced Encryption Standard
DAC: Discretionary Access Control
DSDA: Document Secure Document Deletion Algorithm
FDSPSFSED: Fusion Data Security Protection Scheme for Sensitive E-Documents
HSEA: Hybrid Symmetric Encryption Algorithm
IBE: Identity-Based Encryption Algorithm
KSSM: Key Separation Storage Method
MAC: Mandatory Access Control
PRE: Proxy Re-Encryption Algorithm
RBAC: Role-Based Access Control
RC4: Rivest Cipher 4
UOSMM: User operation state monitoring method

## References

[1] Namasudra S. An improved attribute-based encryption technique towards the data security in cloud computing. Concurrency and Computation: Practice and Experience. 2019;31(3):4364-.

[2] Sun PJ. Privacy Protection and Data Security in Cloud Computing: A Survey, Challenges and Solutions. IEEE Access. 2019;pp(99):1-. doi: 10.1109/ACCESS.2019.2946185

[3] Afrianto I, Heryandi A, Finandhita A, Atin S, editors. Prototype of E-Document Application Based on Digital Signatures to Support Digital Document Authentication. IOP Conference Series: Materials Science and Engineering; 2020: IOP Publishing. 10.1088/1757-899X/879/1/012042

[4] Gai K, Qiu M, Zhao H. Privacy-Preserving Data Encryption Strategy for Big Data in Mobile Cloud Computing. IEEE Transactions on Big Data. 2017:1-. doi: 10.1109/TBDATA.2017.2705807

[5] Bhanot R, Hans R. A review and comparative analysis of various encryption algorithms. International Journal of Security Its Applications. 2015;9(4):289–306.

[6] Chunpeng G, Liu Z, Xia J, Liming F. Revocable Identity-Based Broadcast Proxy Re-encryption for Data Sharing in Clouds. IEEE Transactions on Dependable Secure Computing. 2019;pp(99):1-. doi: 10.1109/TDSC.2019.2899300

[7] Deng H, Qin Z, Wu Q, Guan Z, Zhou Y. Identity-Based Encryption Transformation for Flexible Sharing of Encrypted Data in Public Cloud. IEEE Transactions on Information Forensics Security. 2020;15:3168–80. doi: 10.1109/TIFS.2020.2985532

[8] Li Y, Yu Y, Min G, Susilo W, Ni J, Choo KR. Fuzzy identity-based data integrity auditing for reliable cloud storage systems. IEEE Transactions on Dependable and Secure Computing. 2019;16(1):72–83. doi: 10.1109/TDSC.2017.2662216

[9] Li J, Chen N, Zhang Y. Extended File Hierarchy Access Control Scheme with Attribute Based Encryption in Cloud Computing. IEEE Transactions on Emerging Topics in Computing. 2019:1-. doi: 10.1109/TETC.2019.2904637

[10] Priya A, Tiwari R. A survey: attribute based encryption for secure cloud. International Journal Online of Humanities. 2018;5(3). doi: 10.24113/ojssports.v5i3.70

[11] Sahai A, Waters B. Fuzzy identity-based encryption. Lecture Notes in Computer Science. EUROCRYPT 2005: Advances in cryptology; 2005. Berlin: Springer; 2005.p. 457–73.

[12] Goyal V, Pandey O, Sahai A, Waters B. Attribute-based encryption for fine-grained access control of encrypted data. Proceedings of the 13th ACM Conference on Computer and Communications Security; 2006 Oct 30-Nov 3; Alexandria, VA, USA. New York, NY, USA: Association for Computing Machinery; 2006.

[13] Zhang J, Gao H. A compact construction for non-monotonic key-policy attribute-based encryption. International Journal of High Performance Computing Networking. 2019;13(3):321–30. doi: 10.1504/IJHPCN.2019.098573

[14] Ameri MH, Delavar M, Mohajeri J, Salmasizadeh M. A Key-Policy Attribute-Based Temporary Keyword Search scheme for Secure Cloud Storage. IEEE Transactions on Cloud Computing. 2018;8(3):660–71. doi: 10.1109/TCC.2018.2825983

[15] Taha MB, Talhi C, Ould-Slimane H. Performance Evaluation of CP-ABE Schemes under Constrained Devices. Procedia Computer Science. 2019;155:425–32. doi: 10.1016/j.procs.2019.08.059

[16] Bethencourt J, Sahai A, Waters B, editors. Ciphertext-Policy Attribute-Based Encryption. Proceedings of the 2007 IEEE Symposium on Security and Privacy; 2007. USA: IEEE Computer Society.

[17] Shynu PG, Singh KJ. An Enhanced CP-ABE Based Access Control Algorithm for Point to Multi-Point Communication in Cloud Computing. Journal of Information and Science Engineering. 2017;33(3):837–58. doi: 10.6688/JISE.2017.33.3.15

[18] Li J, Yao W, Han J, Zhang Y, Shen J. User Collusion Avoidance CP-ABE With Efficient Attribute Revocation for Cloud Storage. IEEE Systems Journal. 2018;12(2):1767–77.

[19] Khan F, Hui L, Zhang L, Jian S, editors. An Expressive Hidden Access Policy CP-ABE. IEEE Second International Conference on Data Science in Cyberspace (DSC); 2017; Shenzhen, China.

[20] Mahdi G, Dariush AM, Hassan S. A Thorough Trust and Reputation Based RBAC Model for Secure Data Storage in the Cloud. IEEE Transactions on Parallel Distributed Systems. 2018;30(4):778–88. doi: 10.1109/TPDS.2018.2870652

[21] Batra G, Atluri V, Vaidya J, Sural S. Deploying ABAC policies using RBAC systems. Journal of Computer Security. 2019;27(4):1–24. doi: 10.3233/JCS-191315 31929684 PMC6953980

[22] Cai T, Nie Q, Ouyang K, Zhou J. Role-extended-based RBAC model. Application Research of Computers. 2016;33(3):882–5.

[23] Yu B, Tai X, Ma Z. The study on attribute and trust-based RBAC model in cloud computing. Computer Engineering and Applications. 2020;56(9):84–92. doi: 10.3778/j.issn.1002-8331.1901-0361

[24] Rao KR, Nayak A, Ray IG, Rahulamathavan Y, Rajarajan M. Role recommender-RBAC: Optimizing user-role assignments in RBAC. Computer Communications. 2021;116:140–53. doi: 10.1016/j.comcom.2020.12.006

[25] Joshi M, Joshi KP, Finin T, editors. Attribute based encryption for secure access to cloud based EHR systems. International Conference on Cloud Computing; 2018 July 2–7; San Francisco, CA, USA: IEEE; 2018.

[26] Malviya DK, Lipton M, Verma DRV. A review on secure access to cloud storage by using ABE. International Journal on Future Revolution in Computer Science & Communication Engineering. 2018;4(12):126–31.

[27] Liu G, Pei W, Tian Y, Liu C, Li S. A Novel Conflict Detection Method for ABAC Security Policies. Journal of Industrial Information Integration. 2021;22(2):100200. doi: 10.1016/j.jii.2021.100200

[28] Salama D-D, Abd elkader H, Hadhoud MM. Performance evaluation of symmetric encryption algorithms. International Journal of Network Security. 2008;8(12):280–6.

[29] Mousa A, Hamad A, Applications. Evaluation of the RC4 algorithm for data encryption. International Journal of Computer Science and Applications 2006;3(2):44–56.

[30] Ontrack EasyRecovery. Version 13.0.0.0 [software]. https://www.ontrack.com/en-us/data-recovery/software.

[31] Puran File Recovery. Version 1.2 [software]. http://www.puransoftware.com/File-Recovery.

[32] Wise Data Recovery. Version 5.1.5.333 [software]. https://www.wisecleaner.com/wise-data-recovery.html.

[33] Li R, Dong Z, Zhang Y, Su H, Lang P. Attribute-Based Encryption with Multi-keyword Search. IEEE Second International Conference on Data Science in Cyberspace; June 26–29; Shenzhen, China. IEEE; 2017. p. 172–7.

[34] Wang S, Yao L, Zhang Y, Khan MK. Attribute-based encryption scheme with multi-keyword search and supporting attribute revocation in cloud storage. PLOS ONE. 2018;13(10):e0205675-. doi: 10.1371/journal.pone.0205675 30312345 PMC6185864

[35] Lozupone V. Analyze encryption and public key infrastructure (PKI). International Journal of Information Management. 2018;38(1):42–4. doi: 10.1016/j.ijinfomgt.2017.08.004

[36] Dorman G, inventor; BOX, INC., assignee. File system monitoring in a system which incrementally updates clients with events that occurred in a cloud-based collaboration platform. U.S. patent 9,953,036. 2014 Jul 10.

[37] Subramanya R, inventor; Oracle International Corporation assignee. File change detector and tracker. U.S. patent 9,292,529. 2016 Mar 22.

